# Chromosome Tug of War: Dicentric Chromosomes and the Centromere Strength Hypothesis

**DOI:** 10.3390/cells11223550

**Published:** 2022-11-10

**Authors:** Hunter J. Hill, Kent G. Golic

**Affiliations:** School of Biological Sciences, University of Utah, Salt Lake City, UT 84112, USA

**Keywords:** centromere, dicentric chromosome, kinetochore, CENPA, microtubules, spindle

## Abstract

It has been 70 years since the concept of varied centromere strengths was introduced based on the behavior of dicentric chromosomes. One of the key conclusions from those early experiments was that some centromeres could pull with sufficient force to break a dicentric chromosome bridge, while others could not. In the ensuing decades there have been numerous studies to characterize strengths of the various components involved, such as the spindle, the kinetochore, and the chromosome itself. We review these various measurements to determine if the conclusions about centromere strength are supported by current evidence, with special attention to characterization of *Drosophila melanogaster* kinetochores upon which the original conclusions were based.

## 1. Introduction

The centromere is the chromosomal locus that underlies and encodes the location of the kinetochore. The kinetochore is the multi-protein complex that mediates attachment of the chromosome to spindle microtubules (MT) to provide for proper meiotic and mitotic segregation of homologs and sister chromatids. Centromeres vary considerably in size, suggesting that there could be functional differences between organisms, and possibly even between different chromosomes in the same organism. The centromere/kinetochore/spindle machinery must be sufficiently strong to move a chromosome during normal cell divisions, but all eukaryotes appear to possess centromeres that are considerably stronger than necessary to simply pull chromosomes through cytoplasm. It has been estimated that less than 1 picoNewton (pN) is sufficient to move chromosomes during anaphase [1,2], though higher forces may be present on bioriented chromosomes in metaphase (4–5 pN) [3,4]. However, a single MT:kinetochore may generate 30–65 pN of force [5]—many times what seems to be needed to segregate chromosomes. Higher eukaryotes, with extended centromeres, typically attach multiple microtubules to every kinetochore, potentially allowing for the achievement of much higher forces. This raises several questions about whether there are functional, behavioral, or evolutionary consequences of these differences in centromeres and kinetochores and their attachments to the spindle. For instance: How does centromere size impact chromosome segregation and spindle forces? What are the consequences when different sized centromeres oppose each other in anaphase? Are spindle forces strong enough to break DNA in a dicentric chromosome? How do different centromere sizes evolve?

## 2. Origins: The Centromere Strength Hypothesis

The idea that centromeres could vary in strength, i.e., the pulling force that the associated kinetochore could produce, was proposed by Sears and Câmara [6], based on the behavior of a dicentric chromosome in wheat, and by Novitski [7], based on the behavior of dicentric chromosomes in *Drosophila melanogaster*. It is no coincidence that both of these papers rely on the occurrence of dicentric chromosomes to distinguish strong vs. weak centromeres: as Sears and Câmara put it, “a weak centromere only shows its weakness when opposed by a strong centromere.” Sears and Câmara based their conclusions on direct observation of chromosome behavior in meiotic and mitotic divisions. In Meiosis I, for instance, when the connected centromeres of the dicentric chromosome attached to opposite poles, the dicentric chromosome was usually positioned off the metaphase plate and nearer one pole—that to which the primary, normally located, centromere was attached. Moreover, the entire chromosome most often progressed to the pole of the primary centromere, with the secondary centromere dragged along. Although they could not determine the basis for this strong/weak difference, Sears and Câmara suggested that the rearrangements that generated the dicentric might have split the original centromere unequally to produce the strong and weak centromeres.

**Figure 1:**
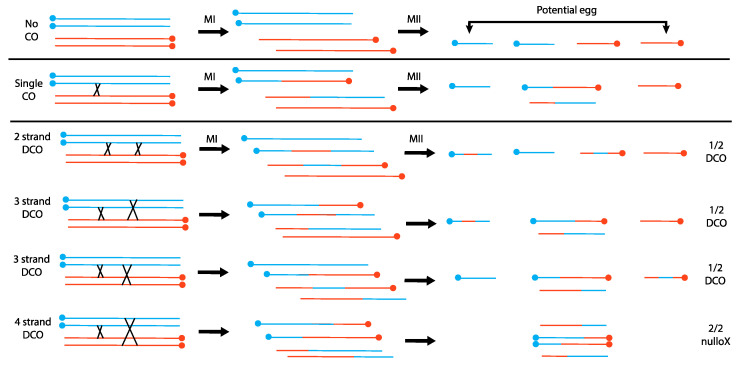
The consequences of crossing over between a normal *X* and an inverted *X* chromosome in *Drosophila melanogaster*. Individual lines are sister chromatids; blue and orange chromosomes are homologs; centromeres are solid circles. In females, the two meiotic divisions occur along a line, and one of the two terminal nuclei becomes the functional egg nucleus. When double crossovers occur, the proportion of embryos that receive a DCO *X* chromosome is 3 × ½ = 3/2; the proportion that receive no *X* is 1 × 2/2 = 2/2. Overall, the ratio of DCO:nullo embryos is 3/2:2/2, or 3:2, which will be the ratio seen among sons.

Novitski’s work also involved competing two different centromeres against each other. To understand Novitski’s results, we must first discuss earlier work of Sturtevant and Beadle in which they examined meiotic crossing over between inverted (In) and normal sequence (+) chromosomes [8] (also recently reviewed by Hawley and Ganetzky [9]). Sturtevant and Beadle observed that In/+ females produced sterile male progeny that carry only the paternal *X* chromosome and no *Y*, called patroclinous males, at a higher rate than would be expected by spontaneous nondisjunction. Chromosomes with double crossovers (DCO) were recovered, but single crossovers (SCO) within the heterozygous inversion, which would generate a dicentric chromosome, were not recovered. It seemed likely that a dicentric chromosome would act as a dominant lethal, yet they did not see an increase in zygotic lethality sufficient to account for the absence of the SCOs [10]. They were able to exclude the trivial explanation that SCOs do not occur within a heterozygous inversion by using an attached-*X* chromosome in which the two *X*’s differed by an inversion. Single exchanges in such a chromosome can produce a circular, or ring, chromosome, and these were recovered. Robbins later confirmed that single exchanges do occur between a normal/inverted pair of *X*’s that are not attached [11]. To explain the lack of zygotic lethality, they reasoned that a single crossover within the inversion would create a dicentric chromosome that connected the exchange chromatids in Meiosis I such that only the nonexchange chromatids, which have a single centromere, would make it to the egg nucleus in Meiosis II (Figure 1; in *Drosophila* females Meiosis I and Meiosis II divisions occur along the same axis, and the innermost terminal nucleus becomes the functional egg nucleus). Although cytogenetic analysis of female meiosis in *Drosophila melanogaster* is challenging, Hinton and Lucchesi were able to confirm the basic tenets of this hypothesis: that dicentric bridges are formed by crossing over and that bridges do not progress to the outer poles in Meiosis II [12]. 

Sturtevant and Beadle’s hypothesis explains both why there is little loss of egg viability due to dicentric formation and the excessive number of patroclinous males. When considering different types of double exchanges, they deduced that 4-strand double exchanges should generate two dicentric chromosomes that would be unable to move to the terminal nuclei, producing nullo-*X* embryos. These nullo-*X* embryos would produce patroclinous sons when fertilized by *X*-bearing sperm. This makes the testable prediction that sons with DCO chromosomes from 2- and 3-strand double exchanges, and patroclinous sons from 4-strand double exchanges, should occur in a 3:2 ratio (Figure 1): this prediction matched their experimental observations. 

**Figure 2:**
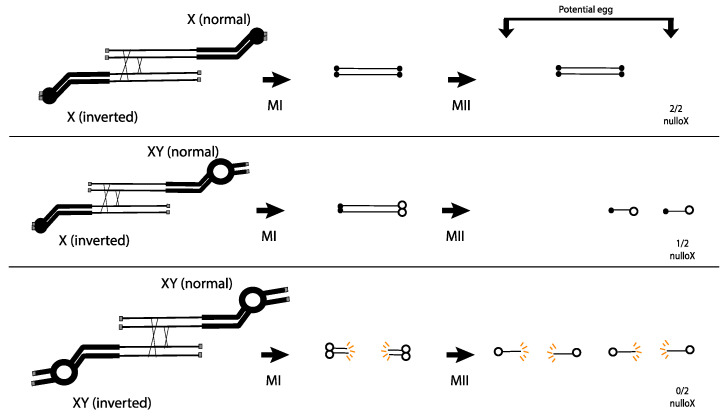
The centromere strength model proposed by Novitski. Only 4-strand DCOs are drawn. Weak (*X*) centromeres are small solid circles, and strong (*Y*) centromeres are large open circles. Grey boxes are telomeres. Acentric products are not shown. Top: Dicentric bridges with two *X* centromeres do not break in MI or MII, and no *X* is transmitted. Middle: Dicentric bridges with one *X* and one *Y* centromere are dragged during MI and segregate during MII, transmitting a lethal dicentric to 50% of the functional egg nuclei. Bottom: Dicentric bridges with two *Y* centromeres break in MI, transmitting broken lethal products to the egg nuclei.

Novitski advanced this work by examining exchanges between inverted and normal sequence chromosomes that differed in their centromere regions. When each homolog carried a normal *X* centromere, Novitski observed the same 3:2 ratio of DCO:patroclinous sons that Sturtevant and Beadle had seen (Figure 2 top). However, if one of the chromosomes was a product of *X-Y* interchange (essentially a translocation between *X* and *Y*), and carried the *X* euchromatin attached to the *Y* centromere and surrounding heterochromatin, the ratio of DCO: patroclinous sons was much closer to 3:1. Novitski proposed that the dicentric chromosomes produced in such females carried centromeres of different strength, and that the stronger centromere was able to drag the weaker centromere to the pole at MI, allowing for its inclusion in the egg nucleus at MII (Figure 2 middle). He presumed that this dicentric chromosome then acted as a dominant lethal. 

This hypothesis was put to the test by examining exchange between inverted and normal sequence chromosomes where each carried a *Y* centromere and surrounding heterochromatin. In this case, the DCO:patroclinous son ratio was approximately 3:0. Novitski interpreted this result as an indication that the *Y* carried the strong centromere, and that, when two strong centromeres pulled against each other in a dicentric at MI, the chromosome bridge broke, delivering a broken chromosome to the egg nucleus where it caused dominant lethality (Figure 2 bottom). This proposal had some basis in the behavior of dicentric chromosomes in plants, where there was extensive evidence for dicentric bridge breakage [13,14,15].

These two sets of experiments, in wheat and in *Drosophila*, can be taken as the first demonstrations that the centromeres, and kinetochores, of different chromosomes, even within a species, could vary in the strength of their pulling force.

## 3. Chromosome vs. Spindle: How Does a Dicentric Break?

One can imagine numerous mechanisms for dicentric chromosome breakage. In plants, breakage has sometimes been attributed to scission by the reforming cell wall during cytokinesis [16]. In organisms without cell walls, the actin–myosin contractile ring might cleave a chromosome during cytokinesis [17]. Alternatively, an endonuclease could cleave a chromatin bridge that might otherwise prevent completion of division [18]. These mechanisms were recently reviewed [19]. In contrast, Novitski’s centromere strength hypothesis implies that the tension exerted on a dicentric chromosome by the kinetochore/spindle assembly breaks the chromatin bridge. Moreover, only some centromere/kinetochore assemblies pull strongly enough to do this, indicating that there must be significant differences between them. Since Novitski’s experiments were based on the behavior of chromosomes in the first meiotic division in female Drosophila, where divisions occur without cytokinesis, breakage of the dicentric cannot be attributed to cytokinesis. It is necessary then to consider whether the tension generated by some, but not all, centromere/kinetochore/spindle combinations is sufficient to break a covalent bond in the phosphodiester–ribose sugar backbone of DNA.

The strength of covalent bonds can be measured directly by atomic force microscopy. Grandbois et al. [20] measured the force required to break a covalent bond as ~1400 picoNewtons (pN) for a S-Au bond and ~2000 pN for a Si-C bond. This difference points to the fact that not all covalent bonds are equal. Notably, these experiments did not measure the covalent bonds that link adjacent nucleotides in DNA. Although C-C bonds appear to be quite strong (estimated at 4000–8000 pN; [20,21]), to our knowledge, the phosphodiester bonds found in the DNA backbone have not been directly tested. When the force required to break dsDNA has been experimentally measured, significantly less force is required. Double strand DNA broke with a force of ~270 pN in one case and ~470 pN in another [22,23].

If a dicentric bridge does break from tension, then the components of the segregation mechanism must be at least as strong, if not stronger, than DNA. The connection between a single spindle fiber and the centrosome requires ~50 pN or more to disrupt [24], though this particular measurement is not relevant to meiotic divisions in Drosophila females, since these divisions are acentriolar [25]. Next, microtubules themselves must convey sufficient tension to the kinetochore without breaking themselves. The tension required to rupture a microtubule has been measured at ~500 pN [26]. Measurements of the strength of the MT:kinetochore coupling vary greatly, from ~9–12 pN in yeast [24,27] to ~50 pN or more in Drosophila [28]. Finally, the connection between the kinetochore and the underlying chromosome must also match or exceed the strength of DNA. To our knowledge, this has not yet been measured. 

Based on these measurements, the weakest point appears to be the connection between MT and kinetochore. However, the strength of this connection can be expected to vary as the numbers of MTs attached to a kinetochore varies. The yeast Saccharomyces cerevisiae has what are termed point centromeres. These are on the order of 120 base pairs in length, each having a defined sequence and encoding a kinetochore which, as mentioned, attaches to a single MT. A kinetochore with a single MT attached should not have sufficient strength to break DNA. However, centromere size and the number of MTs attached to the kinetochore vary over a large range in different eukaryotes [29]. Chromosomes of higher eukaryotes typically have what are called regional centromeres. These can encompass millions of base pairs of DNA and encode kinetochores that attach dozens of MTs: in Drosophila S2 cells, kinetochores are attached to an average of ~11 MTs; human kinetochores are attached to an average of ~17 MTs; in the blood lily, Haemanthus, centromeres may be attached to >100 MTs [28,30,31,32,33,34]. Finally, there are eukaryotes with holocentric chromosomes in which the kinetochore is assembled along the full length of the chromosome. Our discussion below is focused solely on chromosomes with regional centromeres.

The attachment of varied numbers of MTs suggests that the force applied to the kinetochore may also vary. In support of this, Hays and Salmon severed a portion of the microtubules attaching one of the kinetochores of a grasshopper spermatocyte bivalent to its pole [35]. They saw the bivalent move closer to the pole that still had the full complement of intact kinetochore MTs, indicating that metaphase poleward force is at least partly determined by the number of microtubules attached to the kinetochore.

Since force is dependent on the number of MTs attached to the kinetochore, organisms with regional centromeres might have kinetochores that generate force sufficient to break DNA. In Drosophila S2 cells, assuming additive force of multiple kinetochore microtubules, a kinetochore may experience a pulling force of ~135–680 pN, which appears to be within the range required to break DNA [28]. This estimate is nicely matched by a direct measurement of the force that the segregation machinery is capable of exerting on segregating chromosomes in grasshopper spermatocytes. By using a calibrated needle to capture and restrain a segregating chromosome, the force required to stall poleward movement was measured as ~700 pN [36]. However, unresolved questions remain: optical trapping measured the force required to stall anaphase movement of chromosomes in insect spermatocytes as 10 pN or less [3]. Because of the large variation in the estimated or measured strengths of various components, it is not possible to definitively state that the segregation machinery is sufficiently strong to break DNA. Nonetheless, multiple experiments do suggest that the spindle/kinetochore assembly can pull with enough force to break DNA.

## 4. Strong and Weak Centromeres

If the spindle/kinetochore assembly can apply sufficient force to break a dicentric bridge, can this be reconciled with Novitski’s hypothesis that only some centromeres in *Drosophila melanogaster* can exert the needed force? That is, are there actually strong and weak centromeres? 

The centromeres of most eukaryotes are defined by the sites of incorporation of a Histone H3 variant called CENPA (aka CenH3 or Cid). The location of CENPA in a chromosome is invariably correlated with the location of the kinetochore. Furthermore, engineered incorporation of CENPA at a novel site in euchromatin leads to kinetochore assembly at that site [37,38,39,40,41,42,43]. A high level of induced CenpA expression results in its deposition on ectopic sites away from the endogenous centromere. These ectopic CENPA sites also form functional kinetochores [44,45]. CENPA is one of the few chromatin proteins retained on sperm chromosomes during spermiogenesis and is required paternally in *Drosophila* for centromere maintenance across generations [46]. When CENPA is degraded during spermatogenesis (using deGrad-GFP), centromeres of the paternal chromosomes are defective; and when CenpA is overexpressed during spermatogenesis, the centromeres of paternal chromosomes are larger in the next generation. These experiments make it clear that CENPA defines the centromere. 

Certain mouse strains differ significantly in the size of the centromeres that their chromosomes carry [47,48]. In hybrids between two strains where chromosomes differ in centromere size, the chromosomes with the larger centromeres are recovered from females at rates above the Mendelian expectation, a phenomenon termed centromere drive [49]. At Meiosis I, a bivalent that is formed between chromosomes with different sized centromeres tends to reorient on the spindle so that the chromosome with the larger centromere is positioned towards the interior of the embryo—the side that will ultimately give rise to the female pronucleus. When such bivalents attain a stable bioriented attachment, they are not centered between the two spindle poles. Instead, they lie closer to the pole that attaches to the larger centromere. The larger centromere is apparently stronger and pulls the bivalent off center [47,48], supporting the proposition that centromere strength is proportional to centromere size.

If centromere strength is based on centromere size, then Novitski’s hypothesis concerning the behavior of dicentric chromosomes predicts that the *Drosophila melanogaster Y* chromosome should have a larger centromere than the *X*. S2 cells, where Ye et al. made their measurements of forces at the kinetochore, do not carry a *Y* chromosome [28]. However, Raychaudhuri et al. found that the *Y* chromosome has approximately twice as much CENPA as the *X* and the autosomes [46]. In *Drosophila* primary spermatocytes, the *Y* chromosome also attaches significantly more MTs than the *X* chromosome at metaphase of Meiosis I [30]. Based on these results, it seems likely that the centromere of the *Y* chromosome is indeed stronger than the centromere of the *X* chromosome. Given the vagaries of quantitating forces acting on chromosomes, it is not at all unreasonable to imagine that a two-fold difference in centromere size between the *X* and the *Y* could be sufficient to determine whether a dicentric chromosome breaks.

## 5. Reconsidering Novitski

Even though Novitski’s strong/weak centromere hypothesis provides a beautiful explanation of the genetic results, the difficulty of observing female meiotic divisions, and especially live imaging, has prevented direct verification. In our experiments with dicentric chromosomes, we have detected breakage of each of the chromosomes in the *Drosophila melanogaster* genome in mitotic divisions [50,51,52,53,54]. This applies to the *Y*, which should have a strong centromere, and to the *X* and autosomes, which are expected to have weak centromeres. A complication to interpreting these results is that breakage occurred in mitotic divisions where cytokinesis may play a role. 

Despite this, some of our experiments do support Novitski’s hypothesis. In male germline stem cells, sister chromatid exchange (SCE) can be induced in ring-*X* chromosomes to produce a double bridge that can break. After the broken ends are healed by telomere addition, linear derivatives can be recovered [55]. We tested three different ring-*X* chromosomes and found a correlation between the identity of the centromere and the rate of linear chromosome recovery: linear derivatives were recovered more frequently from ring chromosomes with a *Y* centromere (and a large segment of the *Y*) than a ring chromosome with an *X* centromere [56]. This trend was replicated in somatic cells: after inducing SCE, broken chromosomes were detected more frequently if the chromosome had a *Y* centromere than if it had an *X* centromere. If all centromeres and forces were equal between the different ring-*X* chromosomes, either the breakage frequencies should be equal, or the smallest chromosome (that with the *X* centromere) should break most often because the bridge should be stretched the most. We saw the opposite trend: the largest chromosome (with the *Y* centromere) generated linear derivatives frequently and the smallest chromosome very infrequently. This result can be interpreted in the framework of Novitski’s strong/weak centromere hypothesis. The large rings break more frequently because they have *Y*-derived centromeres, and the small ring less frequently owing to its *X*-derived centromere.

**Figure 3:**
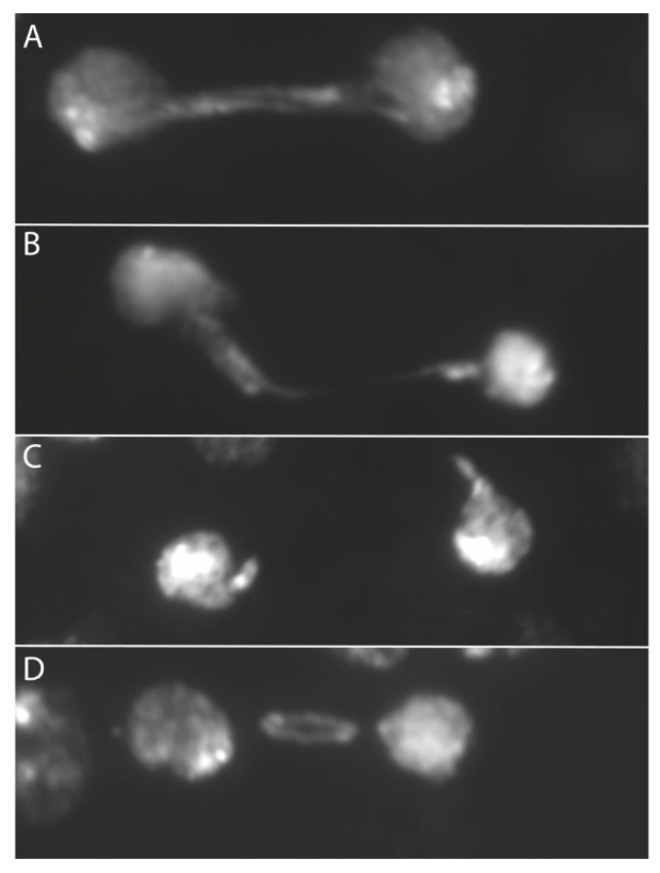
Examples of double dicentric bridges in telophase. Bridges were formed via FLP recombination within a ring chromosome; (**A**) an unbroken double bridge; (**B**) the bottom arm of the double bridge is clearly broken, the other may be intact; (**C**) both arms of the double bridge are broken; (**D**) a lagging bridge.

When we examined anaphase bridges cytologically another relationship emerged: the dicentric produced by SCE in the ring with *X* centromeres could be seen to lag during division more than the ring dicentrics with *Y* centromeres. The term lagging was applied to anaphase bridges where either one or both centromeres were physically separated from the bulk of chromosomes in the daughter nuclei, and tension was apparently lost from the dicentric chromosome (Figure 3). In accordance with the physical measurements, this suggests that the weak point may be the connection between spindle MTs and the kinetochore. This observation further supports the work of Sturtevant and Beadle and of Novitski, both of which suppose that the tension supplied by *X* centromeres is insufficient to break the chromatin bridge of a dicentric chromosome.

There is one circumstance that, on its surface, seems to invalidate the link between strong centromeres and chromosome breakage. In *Drosophila* male Meiosis II, dicentric chromosomes with *Y* centromeres frequently do not break [57] and, like female meiosis, this division occurs without complete cytokinesis. When considered more deeply though, this result would be predicted by Novitski. In Meiosis II, the *Y* kinetochores attach only half the number of MTs as in Meiosis I [30], turning the *Y* centromere into a weak centromere. 

To definitively test the hypothesis that different chromosomes have different strength centromeres, it will be necessary to directly observe the fate of dicentric chromosomes in which the attached centromeres are different from each other. It is very difficult to access female meiosis for visual observation, but mitotic divisions are relatively easy to observe. FLP-mediated sister chromatid fusion is an easy way to generate dicentric chromosomes because it is highly efficient—capable of producing dicentric chromosomes at frequencies that approach 100% [58]. However, these dicentrics join sister chromatids so there is no strong vs. weak competition, but, in *Drosophila*, FLP-mediated recombination between homologous chromosomes can also occur at a significant rate [59]. If the homologs differ by a paracentric inversion, then FLP-mediated recombination between them will generate a dicentric chromosome in which the attached centromeres originate from the homologs, rather than sisters. By using an attached-*XY* chromosome and a normal *X*, the dicentrics that are generated will have different centromeres. It should be possible to study the ensuing competition in mitotic divisions of readily accessible tissues, such as the larval brain or imaginal discs, or in early embryos. It may be possible to visualize winners and losers in a dicentric tug-of-war by seeing which centromeres remain attached and which detach from their poles in such a competition. Such experiments could directly reveal the effects of competition between strong and weak centromeres.

While Novitski’s hypothesis imagines that a dicentric chromosome with two strong centromeres will break, there is an alternative, suggested by Ptashne [60], that could explain his results without requiring chromosome breakage. Although dicentric chromosomes generated in larval brains often break, we have also seen telophase/interphase nuclei with long unbroken bridges or double bridges (produced by SCE in a ring chromosome) stretched between daughter cells, even when these chromosomes carry a *Y* centromere (Figure 3). This extended stretching suggests the possibility that, in female meiosis, strong centromeres are simply more capable of stretching the dicentric bridge. In the case of single exchanges or 3-strand double exchanges, the bridge could retard the movement of the connected centromeres, preventing those centromeres from reaching the egg nucleus during Meiosis II. However, in the case of a 4-strand double exchange, there are no monocentric chromatids—strong pulling might continue to stretch the chromatin bridges and pass a centromere of an unbroken dicentric to the egg nucleus. It seems likely that such a dicentric chromosome would act as a dominant lethal, producing the same outcome as Novitski’s hypothesized chromosome breakage.

## 6. How Did the *Y* Obtain a Large Centromere?

Finally, it is interesting to consider how and why the *Y* chromosome centromere might have evolved to be larger, and potentially stronger, than the other chromosomes in the *Drosophila melanogaster* genome. Centromere drive, a form of meiotic drive based on centromere strength, can explain the advantage that larger, stronger, centromeres have in female meiosis. This drive depends on the fact that only one of the four products of female meiosis is used. Drive occurs because stronger centromeres are able to orient MI spindle attachment and bias their own segregation to the pronucleus [61]. Several other examples of recombination-dependent biased segregation have also been observed in plants and animals. In these cases, exchange between heteromorphic homologs generates asymmetric dyads that influence segregation of the sister chromatids at Meiosis II [62,63,64]. 

Since meiotic recombination does not occur in *Drosophila* males, and all four products of meiosis are routinely functional, these phenomena cannot explain evolution of the large *Y* centromere. However, male meiotic drive systems are common among *Drosophila* species, and generally work by killing sperm that carry the disfavored chromosome [65]. For example, in the Segregation Distorter system, males that are heterozygous for an SD chromosome 2 and an appropriately sensitive homolog transmit SD at rates far above the Mendelian proportion: in many SD/+ genotypes, nearly 100% of the sperm carry the SD chromosome because sperm that receive the SD+ chromosome fail to properly mature after Meiosis [66,67]. Sex ratio systems seem to be particularly prevalent in *Drosophila*, where males carrying an SR *X* chromosome transmit that chromosome at a much higher rate than the Y. In some of these cases, the *Y* is seen to misbehave or mis-segregate in Meiosis II, and this is correlated with the death of sperm destined to receive the *Y* [68,69]. If such a case of sex ratio drive were caused by malfunction of the *Y* centromere, perhaps by binding of a factor that interfered with centromere function, then it is easy to imagine that centromere enlargement might be a mechanism to alleviate the drive. Perhaps the larger *Y* centromere of *Drosophila melanogaster* is a remnant of the adaptive response to such a drive system. 

**Figure 4:**
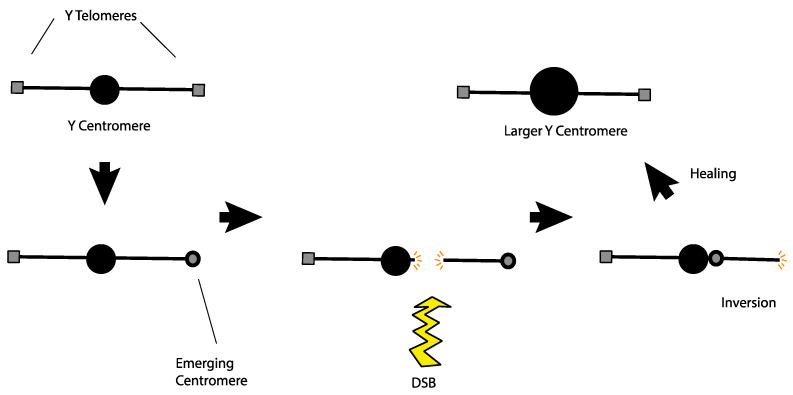
A model to explain how the *Y* centromere may have become enlarged as well as the occurrence of telomeric retrotransposons at the native *Y* centromere. A *Y* chromosome with an ancestral centromere (top left) begins to have centromeric properties emerge at the telomere. Since it is functionally a dicentric chromosome, this configuration will likely result in segregation problems. Those issues may be resolved if a single DSB near the centromere is followed by an inversion distal to the break, bringing the centromeres together. After healing, the result is a normal *Y* chromosome with a bigger centromere at the native locus which would also house telomeric retrotransposons from the ancestral telomeric region.

Alternatively, the large *Y* centromere might have originated as a response to a rogue telomere. Agudo et al. noticed that telomeric retrotransposons are found in the region of the *Drosophila* melanogaster *Y* centromere [70]. They proposed that these telomeric sequences might have developed centromere-like properties at their original location and may have been the ancestral form of the *Y* centromere. Looking at this from a slightly different perspective, if telomeric sequences on the *Y* developed centromere-like properties after the *Y* had already established a functional centromere at a medial position, then the *Y* would become a dicentric chromosome with the attendant difficulties in mitotic and meiotic segregation. An easy escape from this problem would be a chromosomal inversion that moved the terminal neocentromere adjacent to the normal medial centromere to create a single large centromere, solving the segregation problem (Figure 4). This hypothesis even lends itself to experimental tests. Transmissible dicentric chromosomes have been observed in at least two cases in *Drosophila melanogaster* [71,72]. If such a dicentric chromosome were sufficiently detrimental, but not completely lethal, laboratory populations should exhibit strong selection for events that neutralize the segregation problems. Inactivation of one centromere is one mechanism that allows such chromosomes to persist [73]. However, it is also possible that examination of the evolved chromosomes might reveal an enlarged centromere produced by an inversion that merged the two separated centromeres on that chromosome.

## 7. Conclusions

The strong/weak centromere hypothesis was originally proposed by Novitski as a way to explain the outcomes of recombination between inverted and normal sequence chromosomes with *X* or *Y* chromosome-derived centromeres. His experiments, and the preceding work of Sturtevant and Beadle, are beautiful examples of how relatively simple genetic crosses can be used to deduce the behavior of biological systems that defy direct observation.

Novitski’s 70-year-old proposal that the *Y* chromosome of *Drosophila melanogaster* has a strong centromere, while the *X* has a weak centromere, is supported by recent observations that the *Y* carries approximately twice as much CENPA as the *X*. Furthermore, physical measurements of the strength of DNA under tension, and of the force that can be generated by spindle components, generally support the proposition that some kinetochores, but perhaps not all, can pull with sufficient force to break a chromosome. Nonetheless, there is sufficient variation in the measured values to make these conclusions less than certain. 

Our experiments with dicentric chromosomes provide some support for Novitski’s hypothesis that strong *Y* centromeres, pulling on each end of a dicentric bridge, are more likely to break the bridge than are weak *X* centromeres, and include cytological evidence. However, these observations were not in female meiotic divisions. Direct cytological observation of dicentric chromosomes will be required to fully resolve these questions.

## Data Availability

Not applicable.
